# Atlasblockade und Lig.-alare-Läsion – unterschätzt oder übertrieben?

**DOI:** 10.1007/s00482-023-00731-8

**Published:** 2023-07-19

**Authors:** J. Wölfle-Roos

**Affiliations:** 1 Abteilung Orthopädie/Schmerztherapie, m&i Fachklinik Ichenhausen, Krumbacher Str. 45, 89335 Ichenhausen, Deutschland; 2 Universität Ulm, Ulm, Deutschland

**Keywords:** Kopfgelenke, Segmentale Hypomobilität, Hypermobilität, Instabilität, Lig.-alare Ruptur, Upper cervical joints, Segmental hypomobility, Hypermobility, Instability, Alar ligament tear

## Abstract

**Hintergrund:**

Störungen der Kopfgelenke – allen voran die Läsion der Ligg. alaria sowie die Blockierung des Atlas – werden insbesondere in der Laienpresse mit zahlreichen Symptomen assoziiert. Dementsprechend häufig werden Ärzte mit Patienten konfrontiert, die darin eine monokausale Ursache komplexer Beschwerden sehen und auf eine schnelle Lösung hoffen.

**Fragestellung:**

Diese Übersicht stellt die aktuell verfügbare evidenzbasierte Literatur zu Atlasblockade und Lig.-alare-Läsion dar, um ihre Bedeutung differenziert einschätzen zu können.

**Material und Methoden:**

Zusammenfassung und kritische Bewertung einer umfangreichen Literaturrecherche zu Diagnostik, Symptomatik und Therapie der Atlasblockade und Lig.-alare-Läsion.

**Ergebnisse:**

Die Studienlage zeigt, dass *Lig.-alare-Läsionen* nur durch extreme Hochrasanztraumata entstehen und im MRT nur mit mäßiger Reliabilität nachgewiesen werden können. Da zudem in mehreren Studien kein Zusammenhang zwischen Symptomen und Auffälligkeiten im MRT gezeigt werden konnte, ist eine operative Stabilisierung der Kopfgelenke nicht indiziert. Die Vielzahl der Symptome bei *Atlasblockade* kann durch Konvergenz der Afferenzen C1–C3 auf verschiedene Hirnnervenkerne in neuroanatomischen Untersuchungen erklärt werden, der Zusammenhang ist jedoch bisher nicht bewiesen. Erste Studien zeigen eine hochsignifikante Besserung von zervikalen Schmerzen und Bewegungsumfang durch manualtherapeutische Lösung der Blockierung auch 6 Monate nach Behandlung.

**Schlussfolgerung:**

Die Bedeutung der Lig.-alare-Läsion wurde in der Vergangenheit häufig überschätzt, diesbezüglich sollte dem Patienten ein differenziertes, multifaktorielles Krankheitsbild vermittelt werden. Die Atlasblockade ist in erster Linie als mögliche Ursache von Schmerzen und Bewegungseinschränkungen der Halswirbelsäule zu sehen, in diesem Kontext ist manuelle Therapie eine wirksame Option.

## Abstract (en)

**Background:**

Disorders of the upper cervical spine, most notably lesions of the alar ligament and atlas block, are associated with numerous symptoms, especially as reported in the lay press. Thus, physicians are often confronted with patients who see in them a monocausal origin of complex complaints and hope for a quick remedy.

**Objective:**

This review article presents the currently available evidence-based literature on atlas block and alar ligament lesions in order to adequately appreciate their significance.

**Material and methods:**

Summary and critical evaluation of an extensive review of the literature on the diagnostics, clinical presentation, and treatment of disorders of the upper cervical spine.

**Results:**

The current literature shows that alar ligament lesions are caused only by extremely high-speed trauma and that the reliability of their detection on magnetic resonance imaging (MRI) is moderate at best. As several studies have failed to demonstrate a correlation between symptoms and abnormalities of the alar ligaments on MRI, surgical stabilization of the upper cervical joints is not indicated. The diversity of symptoms associated with atlas block may be explained by the convergence of afferent neurons originating in C1–C3 on several cranial nerve nuclei found in neuroanatomical studies, but this association has yet to be proven. First studies show that highly significant improvements in cervical pain and range of motion can be achieved by means of manual therapy of the upper cervical spine with lasting effects even after 6 months.

**Conclusion:**

The importance of alar ligament lesions has often been overrated in the past; however, a more nuanced multifactorial understanding of the disorder should be conveyed to the patient. An atlas block should be considered mainly as a possible cause of pain and restricted range of motion of the cervical spine and in this context manual therapy can be an effective treatment option.

Die Bedeutung der Kopfgelenke polarisiert die Fachwelt und die Laienpresse. Als häufigste Störungen werden die „Atlasblockade“ – die segmentale Hypomobilität der Kopfgelenke [24, 25] –, aber auch die Läsion der Ligg. alaria mit vermuteter Hypermobilität [23] genannt. Die Fülle der Symptome ähnelt sich stark und reicht von Zervikozephalgien über Schwindel und Sehstörungen bis zu Konzentrationsstörungen [1]. Häufig sind Ärzte mit Patienten konfrontiert, die glauben, darin eine monokausale Ursache ihrer komplexen Beschwerden gefunden zu haben. Diese Übersicht stellt die aktuelle evidenzbasierte Literatur dar, um eine differenzierte Einschätzung dieser Störungen vermitteln zu können.

Als Kopfgelenke werden das atlantookzipitale (c0/c1) und atlantoaxiale (c1/c2) Gelenk bezeichnet. Im Atlantookziptalgelenk artikulieren zwei konkave, weitgehend sagittal angeordnete Gelenkflächen des Atlas mit den ebenfalls paarigen ellipsoiden Okzipitalkondylen und ermöglichen hier insbesondere eine Inklinations‑/Reklinationsbewegung mit nur geringer Rotation. Das Atlantoaxialgelenk wird hingegen von dem zapfenartig emporragenden Dens axis dominiert, der zusammen mit dem vorderen Atlasbogen ein sogenanntes Radgelenk bildet und insbesondere Rotationsbewegungen um eine kraniokaudale Achse ermöglicht. Dabei wird der Dens axis von dorsal vom querverlaufenden Lig. transversum atlantis gesichert. Neben dem Lig. transversum kommt auch den paarig angelegten Ligg. alaria eine wichtige Bedeutung zu. Diese ziehen beidseits von der Densspitze flügelartig zu den Okzipitalkondylen (kranialer Anteil) sowie weitgehend horizontal zur Massa lateralis des Atlas (kaudaler Anteil; [20]). Da sich die horizontalen, kaudalen Anteile der Ligg. alaria bei Rotation über dem Dens axis aufspannen, wird durch sie die Rotation begrenzt und moduliert ([20]; Abb. 1).

**Abb. 1:**
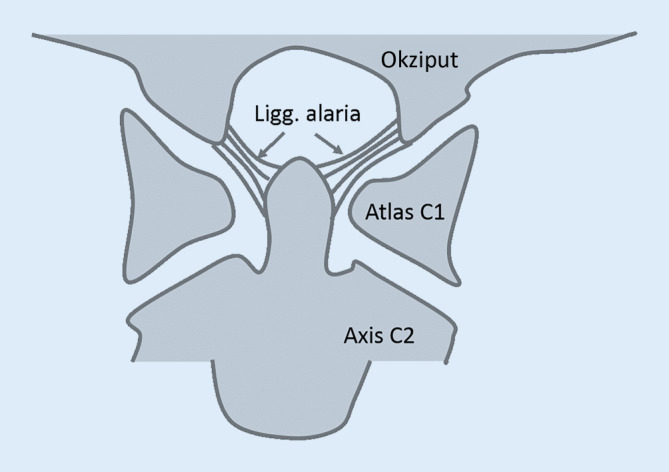
Schematische Darstellung der Kopfgelenke und wichtigsten Bandstrukturen

## „Echte“ Instabilitäten der Kopfgelenke

Insbesondere bei Patienten mit rheumatoider Arthritis, aber auch nach Frakturen von Atlas, Axis und kraniozervikalem Übergang kann es zu einer Instabilität der Kopfgelenke mit sekundärer Myelopathie kommen. Diese „echten“, strukturellen Instabilitäten sind unbedingt ätiologisch und therapeutisch abzugrenzen von der Lig.-alare-Läsion, die allenfalls mit einer Mikroinstabilität assoziiert wird.

Vor dem weitverbreiteten Einsatz von Biologicals war bei Patienten mit rheumatoider Arthritis eine atlantoaxiale Instabilität in 25 % bis 90 % der Fälle nach 10 Jahren Krankheitsdauer zu verzeichnen [15]. Diese wird auf eine Schädigung der Ligg. alaria, des Lig. transversum sowie der Gelenkkapseln durch das entzündliche Pannusgewebe zurückgeführt, wodurch einerseits eine horizontale Subluxation des Dens axis nach dorsal und andererseits eine vertikale Subluxation des Dens axis nach kranial auftreten kann. Nach einer häufig asymptomatischen Initialphase beschreiben die Patienten Schmerzen und/oder Parästhesien im Okzipitalbereich, uncharakteristischen Schwindel sowie Zeichen einer zervikalen Myelopathie mit Gangstörung (häufig als Frühsymptom) und Hyperreflexie [3]. Erst im Verlauf kommen Paresen im Sinne einer Tetraparese hinzu, Hirnnervenstörungen sind sehr selten und treten nur bei ausgeprägter vertikaler Instabilität mit basilärer Impression des Dens axis durch das Foramen magnum auf [3]. Bei Vorliegen einer zervikalen Myelopathie bei rheumatischer atlantoaxialer Instabilität, insbesondere bei intramedullären Signalalterationen im MRT sowie Auffälligkeiten der somatosensorisch evozierten Potenziale (SEP), wird von den meisten Autoren eine operative Versorgung befürwortet. Diese Symptome einer „echten“, strukturellen Instabilität ähneln denen einer vermuteten Mikroinstabilität durch Lig.-alare-Läsion nur wenig, was nahe legt, da diese auf einer völlig unterschiedlichen Pathogenese beruhen.

## Symptome der Lig.-alare-Läsion – strukturelle oder funktionelle Störung?

Im Gegensatz zu den schweren Veränderungen des Kapsel-Band-Apparats, die der rheumatischen oder posttraumatischen Instabilität zugrunde liegen, ist das Ausmaß der beschriebenen Lig.-alare-Läsionen wesentlich geringfügiger. Beobachtet wurden sowohl partielle oder totale Rupturen der Ligg. alaria als auch Strukturveränderungen und Vernarbungen beim intakten Band [22]. Von einigen Autoren wird jedoch auch bezweifelt, dass der mit einer vermeintlichen Lig.-alare-Läsion assoziierte Symptomkomplex überhaupt auf einer strukturellen Schädigung beruht [20], sondern vielmehr eine funktionelle Störung darstellt – also eine Störung ohne Zusammenhang zu einem bildmorphologischen Befund. Dieser Frage soll im Folgenden weiter nachgegangen werden.

### Verursachung von Lig.-alare-Läsionen durch Schleudertrauma unwahrscheinlich

Es wird kontrovers diskutiert, welcher Unfallmechanismus überhaupt geeignet ist, eine Lig.-alare-Läsion herbeizuführen. Obwohl landläufig v. a. in der Laienpresse suggeriert wird, dass jegliche Distorsion der Halswirbelsäule – insbesondere das viel zitierte Schleudertrauma – ausreichend ist für die Lig.-alare-Läsion, ist dieser Umstand durch biomechanische Studien keinesfalls belegt. Die Prädilektionsstelle der Verletzungen bei Schleudertraum ist vielmehr die untere Halswirbelsäule, insbesondere das Facettengelenk c6/7, wie *Panjabi *et al. anhand von acht Halswirbelsäulenpräparaten zeigen konnten, die auf einem Schlitten beschleunigt und dann ruckartig abgebremst wurden. Eine Lig.-alare-Läsion konnte in diesem Versuchsaufbau in keinem der Präparate dargestellt werden [16].

In einem weiteren, ähnlichen Versuchsaufbau von *Maak *et al. wurde an sechs Halswirbelsäulenpräparaten mit Kopfersatzmasse und simulierter paravertebraler Muskulatur die Dehnrate gemessen, die an den Ligg. alaria bei Beschleunigungen von 2 G, 4 G und 8 G wirkt. Diese lag deutlich unter der ebenfalls experimentell ermittelten Dehnrate, die notwendig wäre, um eine Ruptur herbeizuführen [10]. Zudem konnten durch *Castro *et al. an 19 Probanden, die auf einem Schlitten einer ähnlichen Beschleunigung ausgesetzt wurden, keinerlei Strukturveränderungen der Ligg. alaria im Kontrastmittel-MRT nachgewiesen werden [5]. Auch bei einem seitlichen Aufprall konnten *Hartwig* et al. an sechs Halswirbelsäulenpräparaten keine Verletzungen der Ligg. alaria erzeugen [6].

In der einzigen biomechanischen Studie, in der eine Lig.-alare-Läsion experimentell provoziert werden konnte, beinhaltete der Versuchsaufbau eine rotatorische Beschleunigung des Kopfs um eine kraniokaudale Achse mit einem Drehimpuls von über 2 G und Rotation des Kopfs um 60°. Hierbei fanden *Obenauer *et al. in 10 der 19 Präparate eine Lig.-alare-Ruptur, in vier davon mit knöchernem Ausriss an den okzipitalen Kondylen [14]. Da es sich bei diesem Versuchsaufbau um die Simulation eines Hochrasanztraumas handelte, bei dem sich in 18 der 19 Präparate knöcherne oder ligamentäre Verletzungen der oberen Halswirbelsäule zeigten, ist jedoch nicht davon auszugehen, dass eine Lig.-alare-Läsion bei Schleudertrauma regelhaft auftritt. Zudem sollten biomechanische Studien an Leichenpräparaten zurückhaltend bewertet werden, da die biomechanischen Eigenschaften des präparierten Gewebes nicht mit denen vitaler bindegewebiger und muskulärer Strukturen gleichzusetzen sind. Dementsprechend kommt auch die S1-Leitlinie zum Beschleunigungstrauma der Halswirbelsäule zu dem Schluss, dass „eine Verletzung der Ligg. alaria … früher überbewertet [wurde]“ [19]. Darüber hinaus findet die Lig.-alare-Läsion in der Leitlinie keine weitere Erwähnung.

### Lig.-alare-Läsionen im MRT nicht zuverlässig nachweisbar

Genauso umstritten wie die Verursachung der Lig.-alare-Läsion ist ihre Darstellbarkeit in der Bildgebung. Nachdem zunächst mit funktioneller Radiographie und funktioneller Computertomographie versucht wurde, eine Seitendifferenz der Rotation sowie eine paradoxe Rotation zur Diagnosestellung der Lig.-alare-Läsion heranzuziehen [2], konnte schließlich nach flächendeckendem Einsatz des MRT die Ruptur auch direkt dargestellt werden [26].

In der Folge wurde in mehreren Studien die Reliabilität der MR-tomographischen Darstellung der Ligg. alaria untersucht. Zunächst wurde durch *Wilmink *et al. das Ausmaß der Ruptur bzw. der Ausdünnung von 2 verblindeten Radiologen bei 12 Patienten mit persistierenden Beschwerden nach Schleudertrauma sowie 6 asymptomatischen Freiwilligen beurteilt und die Inter- und Intraobserver-Reliabilität bestimmt. Dabei ergab sich eine Interobserver-Reliabilität κ von 0,00 bzw. 0,40 (je nach Auswertung), weshalb nicht von einer ausreichenden Verlässlichkeit bei der Beurteilung von Rupturen ausgegangen wurde [27]. *Krakenes *et al. beschrieben die verblindete Beurteilung eines T2-hyperintensen MRT-Signals der Ligg. alaria durch 3 verschiedene Radiologen bei 92 symptomatischen Patienten nach Schleudertrauma und 30 asymptomatischen Probanden. Hierbei wurde eine Interobserver-Reliabilität κ von 0,49 bis 0,57 beobachtet, weshalb die Verlässlichkeit der MR-tomographischen Beurteilung der Hyperintensität als mäßig eingestuft wurde [8].

Zur weiteren Verbesserung der Diagnosestellung wurden Funktions-MRT-Aufnahmen in ihrer Wertigkeit untersucht. *Pfirrmann *et al. bestimmten bei 50 asymptomatischen Patienten die Differenz der maximalen Rechts- bzw. Linksrotation (3,5 ± 2,8° bei C0/1 und 6,3 ± 4,4° bei C1/2) und verglichen diese mit den von *Antinnes *et al. [2] ermittelten Normalwerten. Da sich bei C0/1 bei 24 % und bei C1/2 sogar bei 31 % der asymptomatischen Probanden pathologische Werte ergaben, wurde auch diese Methode als nicht ausreichend valide eingeschätzt [17]. Eine weitere Funktions-MRT-Methode zur Beurteilung der Ligg. alaria wurde von *Volle *et al. untersucht. Dabei wurden bei 95 symptomatischen Patienten nach Schleudertrauma, bei denen die bisherige Bildgebung nicht konklusiv gewesen war, MRT-Aufnahmen in endgradiger Rotation und endgradiger Seitneigung durchgeführt und in Bezug auf Ruptur bzw. Partialruptur sowie Strukturveränderungen des Bands beurteilt. Die Schwächen dieser Studie liegen in der fehlenden Verblindung, sodass eine Beeinflussung der Befundung durch die bekannte Symptomatik nicht ausgeschlossen werden kann, sowie in der Befundung durch nur einen einzigen Radiologen, weshalb naturgemäß keine Interobserver-Reliabilität bestimmt werden kann. Dementsprechend kann über die Verlässlichkeit dieser Methode bei der Beurteilung der Ligg. alaria keine endgültige Aussage gemacht werden.

Zusammenfassend kann festgehalten werden, dass eine Ruptur bzw. Ausdünnung der Ligg. alaria im MRT nicht mit ausreichender Verlässlichkeit beurteilt werden kann, hyperintense Signalveränderungen hingegen immerhin mit mäßiger Verlässlichkeit.

### MRT-Veränderungen der Ligg. alaria nicht mit Zervikalgien assoziiert

Um den Stellenwert dieser hyperintensen Signalveränderungen der Ligg. alaria im MRT zu überprüfen, wurde von der bereits erwähnten Arbeitsgruppe um *Krakenes* ähnlich wie bei der vorangegangenen Studie [8] verblindet das hyperintense MRT-Signal der Ligg. alaria beurteilt, diesmal an 114 symptomatischen Patienten und 157 asymptomatischen Probanden. Die symptomatischen Patienten entsprachen der WAD-Klassifikation („whiplash-associated disorders“) Grad I und II, wiesen also Zervikalgien ohne neurologische Ausfallserscheinungen auf. Nachfolgend wurde die Prävalenz von hyperintensen Signalveränderungen der Ligg. alaria bei symptomatischen (35,1 %) und asymptomatischen Patienten (30,6 %) verglichen und kein signifikanter Unterschied festgestellt (*p* = 0,434; [21]).

Zu einem ähnlichen Ergebnis kommt die Metaanalyse von *Liu *et al., die fünf ähnliche Studien mit insgesamt 500 Patienten analysierte, in denen das Vorliegen von Zervikalgien WAD I und II mit Auffälligkeiten der Ligg. alaria im MRT korreliert wurde. Zu den MR-tomographischen Auffälligkeiten, die in den fünf Studien beurteilt wurden, zählten sowohl hyperintense Signalveränderungen als auch Hinweise auf eine (Partial‑)Ruptur der Ligg. alaria. Ein statistisch signifikanter Zusammenhang zwischen diesen MRT-Veränderungen und dem Vorliegen von Zervikalgien konnte in den gepoolten Daten dabei nicht nachgewiesen werden (Odds Ratio 1,27, 95 %-Konfidenzintervall 0,87–1,86; [9]).

Weitere Symptome, die im Zusammenhang mit einer Lig.-alare-Läsion beschrieben worden sind, beinhalten Instabilitätsgefühl, Leistungsminderung, Konzentrationsstörungen, Schwindel, Sehstörung, Tinnitus, Parästhesien/Brachialgien, Fallneigung, Übelkeit und Schlafstörungen [23]. Eine belastbare Analyse, ob diese tatsächlich mit Auffälligkeiten der Ligg. alaria im MRT assoziiert sind, fehlt jedoch. In der einzigen Studie, die die Prävalenz dieser Symptome bei Patienten mit und ohne MRT-Veränderungen der Ligg. alaria beschreibt [23], kann aufgrund der fehlenden Verblindung des untersuchenden Radiologen ein Einfluss der geschilderten Symptomatik auf die Beurteilung der MRT-Aufnahmen nicht ausgeschlossen werden. Auffallend ist auch, dass die geschilderten Symptome denen einer „echten“ Instabilität z. B. bei rheumatoider Arthritis nur wenig ähneln, weshalb vermutet werden kann, dass diese Beschwerden nicht Folge einer tatsächlichen Instabilität sind. Denkbar ist eher ein ähnlicher Entstehungsmechanismus der Symptome wie bei der Atlasblockade (s. unten).

Zusammenfassend liegt jedoch kein Nachweis vor, dass das Vorliegen von Symptomen tatsächlich mit Auffälligkeiten der Ligg. alaria im MRT assoziiert ist. Diese fehlende Korrelation zwischen MRT-Befunden und Beschwerden legt nahe, dass der mit einer vermeintlichen Lig.-alare-Läsion assoziierte Symptomkomplex keine strukturelle, sondern eher eine funktionelle Störung darstellt.

### Operative Stabilisierung bei vermuteter Lig.-alare-Läsion nicht indiziert

Bei fehlender zuverlässiger Korrelation zwischen Lig.-alare-Läsion und auslösendem Trauma, unzuverlässiger Nachweisbarkeit der vermuteten Läsion im MRT und fehlender Korrelation zwischen MRT-Befunden und Vorliegen von Symptomen fällt es schwer, eine Grundlage für die Indikationsstellung einer invasiven therapeutischen Maßnahme bei vermuteter Lig.-alare-Läsion zu finden. Dementsprechend gibt es nur eine Studie, die in diesem Fall eine operative Stabilisierung propagiert [22]. Zwar wird in dieser Studie nach Spondylodese C0 auf C2 bei 42 Patienten beschrieben, dass „fast alle Symptome“ nach 5 Tagen verschwunden seien und sich bei allen Patienten das Gleichgewicht verbessert habe. Aufgrund des Fehlens einer Kontrollgruppe, der fehlenden Darstellung, wie es zur Indikationsstellung des Eingriffs kam, der Verwendung einer bildgebenden Methode, deren Reliabilität nicht validiert ist, der fehlenden Angaben, wie die Verbesserung der Symptome evaluiert wurde, und der hohen Komplikationsrate mit Pseudarthrosen in 19 % der Fälle bleibt der Nutzen dieser operativen Maßnahme jedoch äußerst fraglich. Zu diesem Schluss kommt auch eine in der Folge entstandene norwegische Übersichtsarbeit, die unter dem Titel „[Schleudertrauma ist keine Indikation für kraniozervikale Fusion]“ vehement von einer operativen Versorgung abrät [13].

### Fazit Lig.-alare-Läsion: Verunsicherung vermeiden, multifaktorielle Sichtweise stärken

Dem behandelnden Arzt kommt eine Schlüsselfunktion zu bei der frühzeitigen Aufklärung über die extreme Seltenheit der Lig.-alare-Läsion bei Halswirbelsäulendistorsion ohne begleitende knöcherne Läsion sowie über den funktionellen Charakter persistierender Symptome aufgrund des fehlenden Nachweises einer Korrelation zwischen MRT-Auffälligkeiten und Beschwerden. Unbedingt zu vermeiden ist eine weitere Verunsicherung des Patienten durch den Hinweis auf eine vermeintliche Instabilität, durch übertriebene Diagnostik oder sogar Empfehlung einer operativen Versorgung. Stattdessen sollte ein multifaktorielles Krankheitsbild erarbeitet und ein multidisziplinäres, multimodales Therapiekonzept unterstützt werden.

## Atlasblockade – segmentale Hypomobilität der Kopfgelenke

Die mit der Lig.-alare-Läsion assoziierten Symptome ähneln denen stark, die im Zusammenhang mit der sogenannten Atlasblockade – der funktionellen Blockierung der Kopfgelenke – beschrieben wurden. Die Atlasblockade zählt zu den sogenannten Funktionsstörungen, die nicht durch entsprechende bildgebende Maßnahmen wie Röntgen, CT oder MRT sichtbar gemacht werden können, sondern nur durch manualtherapeutische Untersuchung darstellbar sind [7]. Bei der Diagnosestellung ist insbesondere eine segmentale Hypomobilität der Kopfgelenke von Bedeutung, die durch Palpation des Atlasbogens und Processus mastoideus (für C0/1) bzw. des Dornfortsatzes C2 (für C1/2) unter gleichzeitiger Rotation des Kopfs überprüft wird [4]. Bei segmentaler Hypomobilität und gleichzeitigem Vorliegen einer sogenannten „freien Richtung“, in der die Schmerzsymptomatik eine Linderung erfährt, kann die Indikation zur Lösung der Blockierung z. B. mittels Traktionsmanipulation nach Frederick gestellt werden [4]. Dabei sollte über die sehr seltene Komplikation einer Vertebralisdissektion aufgeklärt werden, deren Inzidenz bei 1,3 auf 100.000 Patienten liegt [11, 12].

### Multiple Symptome denkbar durch Konvergenz der C1- bis C3-Afferenz auf Hirnnervenkerne

Durch die Vertreter der manuellen Medizin wurde anhand anatomischer und neurophysiologischer Überlegungen die Hypothese aufgestellt, dass durch die engen Verbindungen zwischen den Afferenzen der Kopfgelenke und den Hirnnervenkernen im Hirnstamm eine Vielzahl von Symptomen erklärt werden kann [7, 24, 25]. Zentraler Bestandteil dieser Hypothese ist der Nachweis der direkten Konvergenz der Afferenzen aus den Gelenkkapseln der Kopfgelenke C0/1 und C1/2 sowie der tiefen kurzen Nackenmuskulatur auf die Hirnnervenkerne III, IV, V, VI, VIII, IX, X und XII. Die damit assoziierten Beschwerden sind in Tab. 1 dargestellt. Über Interneurone auf spinothalamische Bahnen seien auch Störungen höherer Zentren wie Konzentrationsstörungen denkbar, über spinale Interneurone und über den M. longissimus dorsi auch eine Beeinflussung des ISG und der funktionellen Beinlängendifferenz [24, 25]. Ein Nachweis über die Assoziation der Atlasblockade mit den genannten Symptomen steht jedoch bislang aus.

**Tab. 1:** Überblick über die Beschwerden, die durch Verschaltung der Afferenzen der Kopfgelenke mit verschiedenen Hirnnervenkernen assoziiert sein können (nach [24])

Konvergenz auf Hirnnervenkern	Mögliche assoziierte Symptome
N. oculomotorius (III), N. trochlearis (IV), N. abducens (VI)	Unklare Sehstörungen, Doppelbilder
N. trigeminus (V)	Kraniomandibuläre Dysfunktion (CMD), trigeminoautonome Kopfschmerzen, Migräne
N. vestibulocochlearis (VIII)	Schwindel, Tinnitus
N. vagus (X)	Gastrointestinaler Reflux, kardiale Symptome (Hypotonie)
N. glossopharyngeus (IX), N. hypoglossus (XII)	Schluckbeschwerden, Globusgefühl

### Nachweisliche Verbesserung von Schmerz und Bewegungsumfang durch Manualtherapie

Neben der manualtherapeutischen Manipulation zur Lösung der Atlasblockade werden in den sozialen Medien zahlreiche deutlich weniger seriöse Methoden meist mittels speziell dafür entwickelter Apparaturen propagiert und damit Hoffnungen auf eine schnelle, passive und dauerhafte Lösung einer Vielzahl von Symptomen geweckt [1].

In der aktuellen Fachliteratur findet sich jedoch nur eine randomisierte, kontrollierte Studie zur Wirksamkeit der Behandlung der Atlasblockade durch manuelle Therapie. *Rodriguez-Sanz *et al. untersuchten 58 Patienten mit Hypomobilität der Kopfgelenke, von denen 29 Patienten 4 Wochen lang einem täglichen Eigentrainingsprogramm folgten, das bei den anderen 29 Patienten von zusätzlichen manualtherapeutischen Manipulationen C0/1, C1/2 und C2/3 einmal pro Woche begleitet wurde. In der Nachuntersuchung 1, 3 und 6 Monate nach Beendigung der Intervention zeigten sich noch immer hochsignifikant bessere Werte der manualtherapeutisch behandelten Patienten im Vergleich zur Kontrollgruppe in Bezug auf Schmerzniveau, Bewegungsumfang und Neck Disability Index. Beispielsweise konnte der Schmerz auf der visuellen Analogskala (VAS) bei den Patienten der Interventionsgruppe im Vergleich zur Kontrollgruppe bei einem Ausgangswert von 3,36 ± 1,97 (vs. 3,76 ± 2,53 Kontrollgruppe) nach 1 Monat auf 0,75 ± 1,42 gesenkt werden (vs. 2,89 ± 2,44, *p* < 0,016) mit anhaltender Schmerzlinderung nach 3 Monaten mit 0,80 ± 1,30 (vs. 3,87 ± 2,71, *p* < 0,001) und nach 6 Monaten mit 0,98 ± 1,49 (vs. 3,91 ± 2,84, *p* < 0,001; [18]). Das Vorliegen weiterer Symptome oder gar die Besserung dieser Symptome unter manualtherapeutischen Maßnahmen wurde dabei nicht untersucht. Diese Studie stellt dennoch einen belastbaren Nachweis der Wirksamkeit der manuellen Medizin zur Behandlung der Atlasblockade dar. Ein direkter Rückschluss auf die Wirksamkeit anderer, in den sozialen Medien angebotener Maßnahmen zur Lösung einer Atlasblockade lässt sich daraus jedoch nicht ziehen. Eingeschränkt wird die Aussage der Arbeit dadurch, dass aufgrund der fehlenden Verblindung in Bezug auf die Intervention ein Placeboeffekt nicht ausgeschlossen werden kann.

### Fazit Atlasblockade: Symptome nicht überbewerten, manuelle Therapie als Option

Die Atlasblockade sollte vom behandelnden Arzt in erster Linie als mögliche Ursache von Zervikalgien, zervikogenen Zephalgien sowie Bewegungseinschränkungen der Halswirbelsäule vermittelt werden, in diesen Fällen ist auch die manuelle Therapie eine wirksame Therapieoption. Aufgrund fehlender Studienergebnisse ist von anderen, insbesondere kommerziellen Methoden zur Lösung der Atlasblockade abzuraten. In Bezug auf die Vielzahl weiterer Symptome wie Tinnitus, Schwindel, Konzentrationsstörungen etc. sollte die Atlasblockade im Rahmen eines multifaktoriellen Krankheitsbilds als eine zwar bisher nicht bewiesene, jedoch theoretisch mögliche Ursache verstanden werden.

## Fazit für die Praxis

- Die Symptome einer „echten“ Instabilität der Kopfgelenke, z. B. bei rheumatoider Arthritis, bestehen in okzipitalen Schmerzen und Parästhesien sowie Zeichen einer zervikalen Myelopathie mit dem führenden Frühsymptom der Gangstörung und ähneln denen einer Atlasblockade oder Lig.-alare-Läsion nur wenig.
- Der Stellenwert einer Lig.-alare-Läsion wird häufig überschätzt. MR-tomographische Auffälligkeiten der Ligg. alaria haben nur wenig Handlungskonsequenz, da eine Läsion nur unzuverlässig nachgewiesen werden kann und zudem kein Zusammenhang zu klinischen Symptomen gezeigt wurde. In diesen Fällen ist von einer operativen Stabilisierung dringend abzuraten. Stattdessen sollte eine weitere Verunsicherung durch übertriebene Diagnostik vermieden und ein multifaktorielles Krankheitsbild vermittelt werden.
- Die Atlasblockade ist in erster Linie als mögliche Ursache von Schmerzen und Bewegungseinschränkungen der Halswirbelsäule zu sehen, in diesem Kontext ist manuelle Therapie ein wirksame Option. Zwar sind vielfältige weitere Symptome aufgrund der Konvergenz der Afferenzen der Kopfgelenke auf verschiedenste Hirnnervenkerne anatomisch/neurophysiologisch denkbar, jedoch bisher nicht nachgewiesen.
